# Formation of macrocyclic lactones in the Paternò–Büchi dimerization reaction

**DOI:** 10.3762/bjoc.7.35

**Published:** 2011-02-28

**Authors:** Junya Arimura, Tsutomu Mizuta, Yoshikazu Hiraga, Manabu Abe

**Affiliations:** 1 Department of Chemistry, Graduate School of Science, Hiroshima University (HIRODAI), 1-3-1 Kagamiyama, Higashi-Hiroshima, Hiroshima 739-8526, Japan; 2 Japan Science and Technology Agency, CREST, 5 Sanbancho, Chiyodaku, Tokyo, 102-0075, Japan

**Keywords:** furans, macrocyclic lactone, oxetane, Paternò–Büchi reaction, photochemical reaction

## Abstract

Furan-2-ylmethyl 2-oxoacetates **1a**,**b**, in which the furan ring and the carbonyl moiety were embedded intramolecularly, were synthesized from commercially available furan-2-ylmethanol and their photochemical reaction (*h*ν > 290 nm) was investigated. Twelve-membered macrocyclic lactones **2a**,**b** with *C_i_* symmetry including two oxetane-rings, which are the Paternò–Büchi dimerization products, were isolated in ca. 20% yield. The intramolecular cyclization products, such as 3-alkoxyoxetane and 2,7-dioxabicyclo[2.2.1]hept-5-ene derivatives, were not detected in the photolysate.

## Findings

**Scheme 1:**
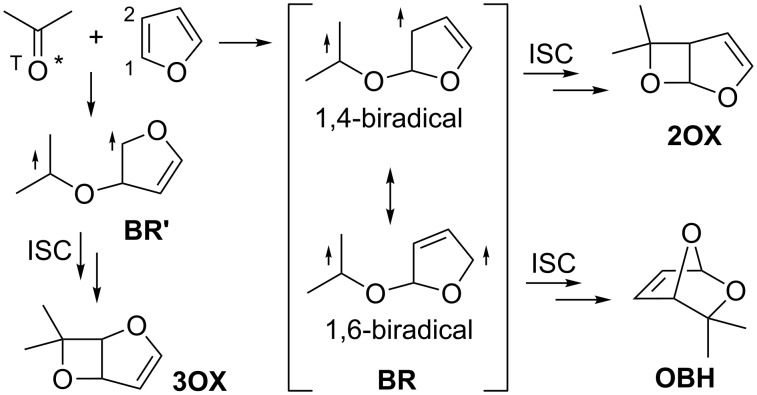
Reaction of furan with triplet excited carbonyls, regioselectivity.

Photochemical [2 + 2] cycloaddition reaction of alkenes with carbonyls, so-called Paternò–Büchi reaction [1–13], is one of the most efficient methods for preparing synthetically useful four-membered heterocyclic compounds, i.e., oxetanes. The Paternò–Büchi reaction of furan with a triplet carbonyl, such as n,π* triplet benzophenone, produces regioselectively 2-alkoxyoxetanes **2OX** (Scheme 1). The regioselective formation is rationalized by the relative stability of the intermediary triplet biradicals, **BR** versus **BR’**, and also by the relative nucleophilicity of the furan-ring carbons, i.e., C1 versus C2 (Scheme 1) [14–18].

**Scheme 2:**
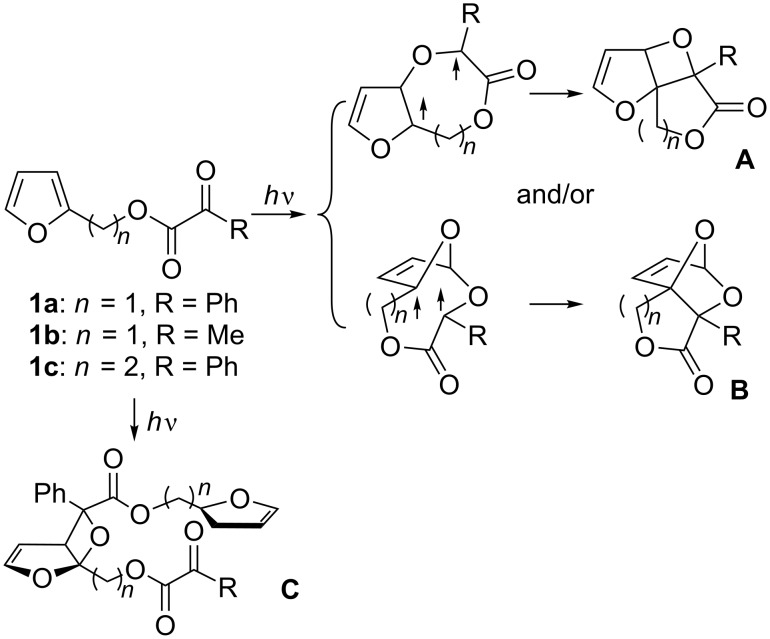
Possible pathways for the photochemical reaction of furan derivatives **1a–c**.

Biradical **BR**, in principle, possess two resonance forms, i.e., 1,4-biradical form and 1,6-biradical form. The 1,4-biradical form affords oxetane **2OX** after the intersystem crossing (ISC). Alternatively, 2,7-dioxabicyclo[2.2.1]hept-5-ene **OBH** would be formed from the 1,6-biradical form. The regioisomeric oxetane **3OX** should be formed via the regioisomeric biradical **BR’**. Biradical **BR** is energetically more stable than **BR’**, because **BR** can undergo radical delocalization. The electrophilic oxygen of the excited carbonyl should preferably interact with more nucleophilic C1 carbon to give selectively the biradical **BR**. Thus, only the 2-alkoxyoxetane **2OX** has been observed in the Paternò–Büchi reactions reported so far [19–27]. Thus, in this study, furan-2-ylmethyl 2-oxoacetates **1a**,**b** and 2-(furan-2-yl)ethyl 2-oxo-2-phenylacetate **1c** [28] were synthesized, in which the furan ring and the carbonyl moiety are connected intramolecularly, and their photochemical reactions were investigated to see whether the reaction proceeds intramolecularly to produce the 3-alkoxyoxetane derivative **A** and/or the dioxabicyclo[2.2.1]hept-5-ene derivative **B**, or intermolecularly to give the 2-alkoxyoxetane derivative **C** (Scheme 2).

**Scheme 3:**
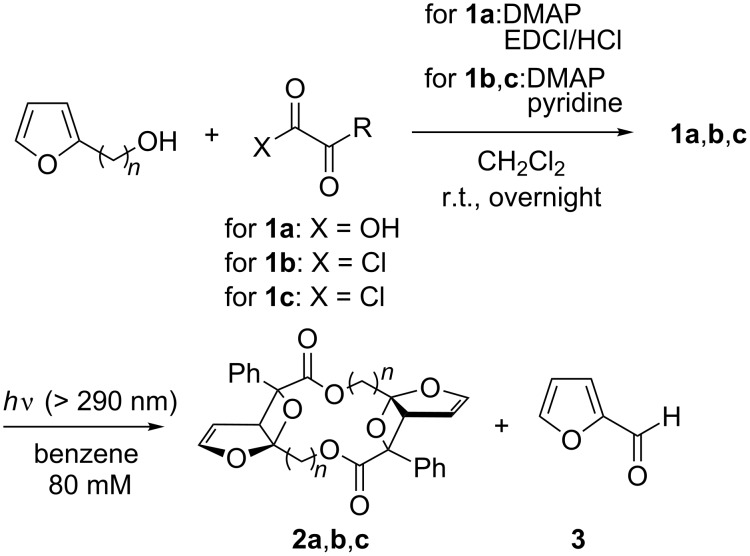
Synthesis and the photochemical reaction of furan-2-ylmethyl 2-oxoacetates **1a**,**b**.

**Figure 1:**
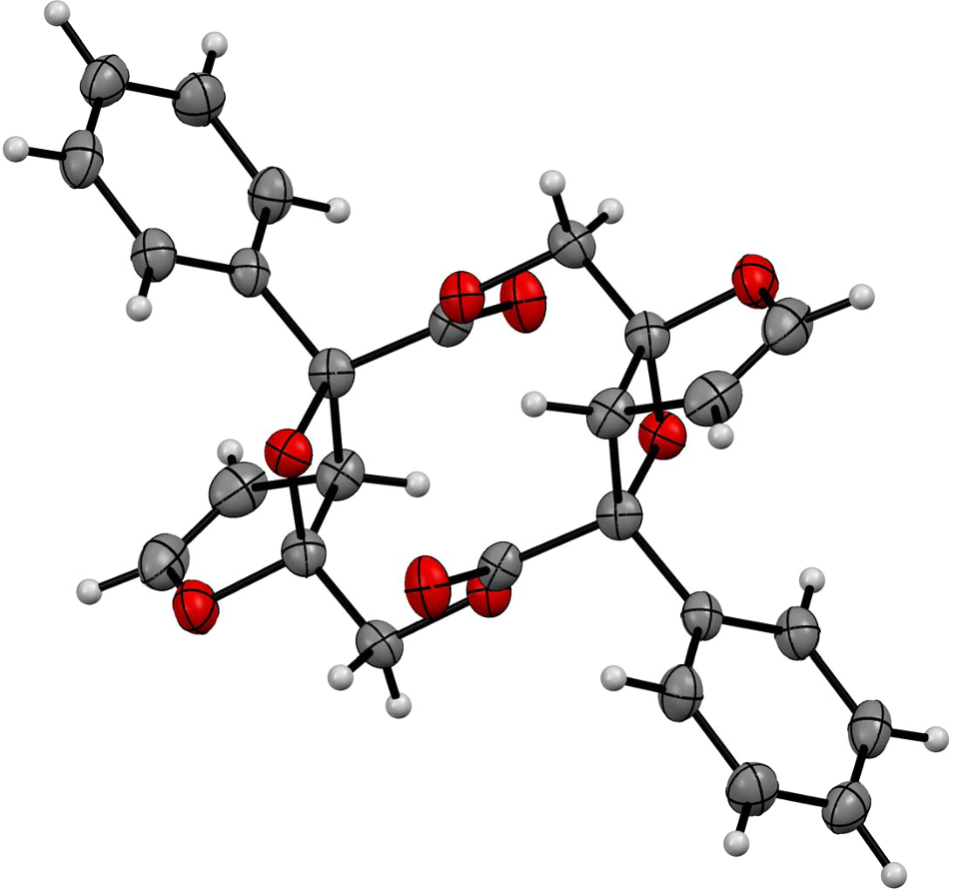
X-ray crystal structure of the macrocyclic lactone **2a**.

**Figure 2:**
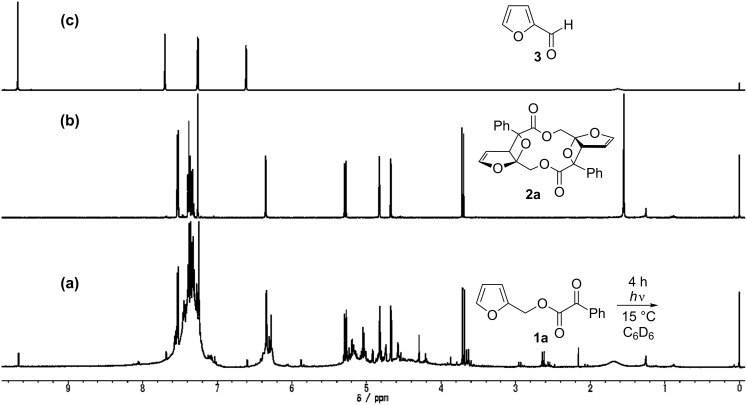
^1^H NMR spectra (500 MHz) for (a) the photolysate of **1a** after 4 h irradiation in degassed and dried C_6_D_6_ solution, (b) for isolated macrocyclic lactone **2a**, and (c) for furan-2-carbaldehyde (**3**).

Compounds **1a**–**c** [28] were synthesized from furan-2-ylmethanol or furan-2-ylethanol [29] (Scheme 3). Compound **1a** (R = Ph) was irradiated in degassed benzene with a high-pressure Hg lamp with a Pyrex filter (Scheme 3). Interestingly, the Paternò–Büchi dimer product **2a** (R = Ph), which possesses *C_i_* symmetry, was obtained as the major product and contains the biologically important macrocyclic lactone structure [30–33]. The structure of **2a** was unequivocally determined by the X-ray crystallographic analysis (Figure 1). The one-step preparation of the highly functionalized twelve-membered macrocyclic lactone is synthetically attractive. Intramolecular products, such as compounds **A** and **B**, were not detected in the photolysate, although intramolecular cyclization products are known to be products in the photoreaction of 3-substituted furan derivatives [11,21]. Furan-2-carbaldehyde (**3**) was the only assignable product during the photochemical reaction, which was monitored by ^1^H NMR spectroscopy (Figure 2). The intermolecular Paternò–Büchi reaction product, i.e., **C** in Scheme 2, was also not observed in the photolysate. This result suggests that the intramolecular Paternò–Büchi reaction of **C** is faster than the first intermolecular Paternò–Büchi reaction of **1a**. The photoreaction of **1b** (R = Me) gave **2b** and **3** in 25% and 18%, respectively (Scheme 3). The dimerization product **2c** was not observed in the reaction of **1c**. Only polymeric products were present in the photolysate. Although the dimerization is sensitive to the chain-length, the Paternò–Büchi dimerization reaction could in future be applicable to the synthesis of a variety of macrocyclic lactones.

To investigate the effects of concentration, solvent, and temperature on the formation of **2a**, the photochemical reaction of **1a** was conducted under the variety of conditions (Table 1). The yield of intermolecular product **2a** was expected to be improved when the concentration of **1a** was increased; however, no concentration effect was observed (entry 2). Under the reaction conditions, the formation of polymeric products increased as evidenced by ^1^H NMR analysis of the photolysate. Even with low concentrations of **1a** (entry 3), the intramolecular photoproducts **A** and **B** were not detected by ^1^H NMR (500 MHz). To investigate the medium effect on the formation of **2a**, the photochemical reaction of **1a** was performed in several solvents (entries 4–6). The yield of **2a** decreased with increasing solvent polarity; 16% in toluene (entry 4) and 10% in CH_3_CN (entry 6). Temperature had no effect on the yield of **2a** (entries 7–9).

**Table 1:** Formation of **2a** in the photochemical reaction of **1a** under the various conditions^a^.

entry	solvent	concentration of **1a** (mM)	temperature (°C)	yield of **2a** (%)^b^

1	benzene	80	15	18
2	benzene	800	15	8
3	benzene	8	15	17
4	toluene	80	15	16
5	CH_2_Cl_2_	80	15	10
6	CH_3_CN	80	15	10
7	benzene	80	50	5
8	toluene	80	−35	18
9	toluene	80	−75	14

^a^The photochemical reactions of **1a** were performed for 4 h with a high-pressure Hg lamp (300 W) with a Pyrex filter under a dry nitrogen atmosphere in dried and degassed solvent. ^b^The yields of **2a** were determined on the basis of ^1^H NMR (500 MHz) peak areas; error ± 3%. Dimethyl fumarate was used as an internal standard.

In summary, the intramolecular products such as **A** and **B** were not observed in the photochemical reaction of furan derivatives **1a**,**b**, but interestingly the Paternò–Büchi dimers **2a**,**b** with the *C_i_* symmetry, i.e., macrocyclic lactones, were isolated in ca. 20% yield. The results indicate that the intramolecular reactions, which produce 3-alkoxyoxetanes and 2,7-dioxabicyclo[2.2.1]hept-5-ene (Scheme 1 and Scheme 2), are slower than the intermolecular reaction which leads to the preferential formation of 2-alkoxyoxetane **C** which is followed by a second Paternò–Büchi reaction to give the observed macrocyclic lactones **2**. This finding should stimulate future experimental and computational studies on the mechanistically and synthetically fascinating formation of macrocyclic lactone derivatives.

## Experimental

NMR and MS measurements were made using JEOL JMN-LA500 and Thermo Fisher Scientific LTD Orbitrap XL spectrometers, respectively, at the Natural Science Center for Basic Research and Development (N-BARD), Hiroshima University.

The furan derivatives **1a**–**c** (129 mg, 0.561 mmol) were dissolved in benzene (7.0 ml) and the degassed reaction mixture was irradiated with a high-pressure Hg lamp (300 W, *h*ν > 290 nm) with a Pyrex filter. After 13 h, the solvent was removed in vacuo and dimethyl fumarate added as an internal standard. ^1^H NMR (500 MHz, CDCl_3_) was measured to determine the ratio of products. After the photoreaction, the residue was purified by repeated column chromatography and PTLC (hexane–EtOAc = 2:1) to give **2a**,**b** as colorless crystals.

**2a**: ^1^H NMR (500 MHz, CDCl_3_) δ 7.55–7.51 (m, 4H), 7.40–7.31 (m, 6H), 6.35 (dd, *J* = 3.1, 1.1 Hz, 2H), 5.28 (dd, *J* = 12.5, 0.9 Hz, 2H), 4.82 (dt, *J* = 3.0, 0.9 Hz, 2H), 4.67 (dd, *J* = 3.0, 1.1 Hz, 2H), 3.71 (d, *J* = 12.5 Hz, 2H); ^13^C NMR (125 MHz, CDCl_3_): δ 171.6 (C), 148.7 (CH), 136.0 (CH), 128.6 (CH), 128.4 (CH), 125.7 (CH), 112.6 (C), 102.1 (CH), 90.7 (C), 61.4 (CH2), 55.2 (CH); HRMS (ESI) *m/z* calcd for C_26_H_20_O_8_Na (M + Na)^+^ 483.10504, found 483.10526.

**2b**: ^1^H NMR (500 MHz, CDCl_3_): δ 6.66 (dd, *J* = 3.0, 1.2 Hz, 2H), 5.23 (dt, *J* = 3.0, 0.8 Hz, 2H), 5.14 (dd, *J* = 12.5, 0.8 Hz, 2H), 4.27 (dd, *J* = 3.0, 1.2 Hz, 2H), 3.77 (d, *J* = 12.5 Hz, 2H), 1.57 (s, 6H); ^13^C NMR (125 MHz, CDCl_3_): δ 173.3 (C), 149.8 (CH), 112.7 (C), 101.3 (CH), 88.1 (C), 61.5 (CH_2_), 53.1 (CH), 21.2 (CH_3_); HRMS (ESI) *m/z* calcd for C_16_H_16_O_8_Na (M + Na)^+^ 359.07374, found 359.07391.

## Supporting Information

File 1 Experimental section for preparation of compounds **1a**–**c**, the detail of the X-ray structure of compound **2a**, and ^1^H NMR and ^13^C NMR spectra for compounds **2a**,**b**.

File 2 X-Ray crystallographic data for compound **2a**.
