# Supplementary material for: Formation of macrocyclic lactones in the Paternò–Büchi dimerization reaction
Source: Beilstein J Org Chem. 2011 Feb 28;7:265–9. doi: 10.3762/bjoc.7.35 (PMC3063007; doi:10.3762/bjoc.7.35)
Supplement: File 1 — Experimental section for preparation of compounds 1a–c, the detail of the X-ray structure of compound 2a, and 1H NMR and 13C NMR spectra for compounds 2a,b. [file Beilstein_J_Org_Chem-07-265-s001.pdf]

Supporting Information

for

Formation of macrocyclic lactones in the Paternò–Büchi dimerization reaction

Junya Arimura[1], Tsutomu Mizuta[1], Yoshikazu Hiraga[1], and Manabu Abe*[1,2]

Address: [1]Department of Chemistry, Graduate School of Science, Hiroshima University (HIRODAI), 1-3-1 Kagamiyama, Higashi-Hiroshima, Hiroshima 739-8526, Japan and [2]Japan Science and Technology Agency, CREST, 5 Sanbancho, Chiyodaku, Tokyo, 102-0075, Japan

Email: Manabu Abe - mabe@hiroshima-u.ac.jp

* Corresponding author

Experimental section for preparation of compounds 1a–c, the detail of the X-ray structure of compound 2a, and ^{1}H NMR and ^{13}C NMR spectra for compounds 2a,b.

Contents:

1. General Method S2

2. Preparation of the substrates **1a**–**c** S2–S4

3. Photoreaction of **1a**,**b** S4,S5

4. X-Ray Data of the dimeric product **2a** S5,S6

5. ^{1}H and ^{13}C NMR spectra S7,S8

6. References S9

General Method: All reagents were obtained from commercial suppliers and used without further purification. All solvents were distilled over CaH_2 before use. Merck silica gel $60F_{254}$ TLC aluminum sheets were used for routine monitoring of reactions. Column chromatography was performed on Merck silica gel 60 (70-230 mesh, ASTM). Merck silica gel $60F_{254}$ was used for preparative thin-layer chromatography. NMR and MS measurements were made using JEOL JMN-LA500 and LTQ Orbitrap XL, respectively, at the Natural Science Center for Basic Research and Development (N-BARD), Hiroshima University.

Furan-2-ylmethyl 2-oxo-2-phenylacetate (1a). To a stirred solution phenylglyoxylic acid (1.59 g, 10.6 mmol) in CH_2Cl_2, was added DMAP (1.24 g, 10.1 mmol) and furfuryl alcohol (1.03 g, 10.5 mmol) at 0°C. The mixture was stirred at 0°C for 15min and EDCI HCl (4.19 g, 21.8 mmol) added to the reaction mixture at 0°C, which was then stirred overnight at r.t. The reaction was quenched with water and the product was extracted with CH_2Cl_2. The organic layer was washed with water, then brine, dried over Na_2SO_4, and concentrated in vacuo. The residue was purified by column chromatography (hexane : EtOAc = 4 : 1) to give **1a** as a yellow oil (701 mg, 29%).

^{1}H NMR (500 MHz, $CDCl_3$) δ 7.97 (dd, *J* = 8.5, 1.2 Hz, 2 H), 7.68-7.62 (m, 1 H), 7.56-7.44 (m, 3 H), 6.54 (d, *J* = 3.4 Hz, 1 H), 6.40 (dd, *J* = 3.4, 1.8 Hz, 1 H), 5.37 (s, 2 H).

2-(Furan-2-yl)methyl 2-oxo-2-methyllacetate (1b). To stirred pyruvic acid (1.03 g, 11.7 mmol) was added $MeOCHCl_2$ (1.00 ml, 11.1 mmol) at 0 °C. The mixture was stirred at 50 °C for 30 min. The byproduct (methyl formate) was removed by evaporation to give 2-oxopropanoyl chloride as a yellow oil which was not further purified. To a stirred solution furfuryl alcohol (822 mg, 8.38 mmol) and pyridine (0.820 ml, 10.1 mmol) in CH_2Cl_2, was added 2-oxopropanoyl chloride (883 mg, 8.29 mmol) and DMAP (108 mg, 0.881 mmol) at 0 °C. The mixture was stirred overnight at r.t. The reaction was quenched with water and the product extracted with CH_2Cl_2. The organic layer was washed with water, then brine and dried over Na_2SO_4. The solvent was removed by evaporation. The residue was purified by column chromatography (hexane : EtOAc = 2 : 1) to give **1b** as a yellow oil (617 mg, 44%). ^{1}H NMR (500 MHz, $CDCl_3$) δ 7.44 (dd, *J* = 1.8, 0.6 Hz, 1H), 6.49 (d, *J* = 3.3 Hz, 1H), 6.38 (dd, *J* = 3.3, 1.8 Hz, 1H), 5.23 (s, 2H), 2.47 (s, 3H); ^{13}C NMR (125 MHz, $CDCl_3$) δ 191.3 (C), 160.3 (C), 147.9 (C), 143.8 (CH), 111.9 (CH), 110.7 (CH), 59.5 (CH_2), 26.7 (CH_3); IR (film, cm^{-1}) 3151, 3127, 3016, 2961, 2927, 1734, 1295, 1157, 1018; HRMS (ESI) *m/z* calcd for $C_8H_8O_4Na$ $(M + Na)^+$ 191.03148, found 191.03174.

2-(Furan-2-yl)ethyl 2-oxo-2-phenylacetate (1c). To a stirred phenylglyoxylic acid (1.36 g, 9.05 mmol) in CH_2Cl_2, was added $MeOCHCl_2$ (0.820 ml, 9.06 mmol) at 0 °C. The mixture was stirred at 50 °C for 90 min. The solvent and methyl formate (byproduct) were removed by evaporation to give phenylglyoxylyl chloride as a yellow oil which was not further purified. To a stirred solution 2-(furan-2-yl)ethanol [1] (505 mg, 4.50 mmol) and pyridine (0.450 ml,

5.56 mmol) in CH_2Cl_2, was added phenylglyoxylyl chloride (1.12 g, 6.64 mmol) and DMAP (67.7 mg, 0.554 mmol) at 0 °C. The mixture was stirred overnight at r.t. The reaction was quenched with water and the product was extracted with CH_2Cl_2. The organic layer was washed with water, then brine and dried over Na_2SO_4. The solvent was removed by evaporation. The residue was purified by column chromatography (hexane : EtOAc = 4 : 1) to give **1c** as a yellow oil (971 mg, 88%). ^{1}H NMR (500 MHz, $CDCl_3$): δ 7.94 (dd, *J* = 8.2, 0.9 Hz, 2H), 7.65 (tt, *J* = 7.5, 1.2, 1H), 7.49 (t, *J* = 5.3, 2H), 7.35 (dd, *J* = 1.8, 0.8 Hz, 1H), 6.32 (dd, *J* = 3.2, 1.8 Hz, 1H), 6.14 (dd, *J* = 3.2, 0.7 Hz, 1H), 4.64 (t, *J* = 6.8, 2H), 3.14 (t, *J* = 6.8, 2H); ^{13}C NMR (125 MHz, $CDCl_3$) δ 186.1 (C), 163.6 (C), 150.9 (C), 141.8 (CH), 134.9 (CH), 132.4 (C), 130.1 (CH), 128.9 (CH), 110.4 (CH), 107.0 (CH), 63.8 (CH_2), 27.6 (CH_2); IR (film, cm^{-1}) 3150, 3122, 3064, 2966, 2907, 1740, 1690, 1200, 1176, 1002; HRMS (ESI) *m/z* calcd for $C_{14}H_{12}O_4Na$ $(M + Na)^+$ 267.06278, found 267.06296.

Photoreaction of 1a. The furfuryl alcohol derivative **1a** was dissolved in benzene (80 mM) and the degassed reaction mixture irradiated with a high-pressure Hg lamp (300 W, *h*ν > 290 nm) with a Pyrex filter. After 13 h, solvent was removed in vacuo and dimethyl fumarate added as an internal standard. ^{1}H NMR (500 MHz, $CDCl_3$) was measured to determine the ratio of products. After the photoreaction, the residue was purified by repeated column chromatography and PTLC (hexane : EtOAc = 2 : 1) to give **2a** as colorless crystals. m.p. 151 °C (dec); ^{1}H NMR (500 MHz, $CDCl_3$) δ 7.55–7.51 (m, 4H), 7.40–7.31 (m, 6H), 6.35 (dd, *J* = 3.1, 1.1 Hz, 2H), 5.28 (dd, *J* = 12.5, 0.9 Hz, 2H), 4.82 (dt, *J* = 3.0, 0.9 Hz, 2H), 4.67 (dd, *J* = 3.0, 1.1 Hz, 2H), 3.71 (d, *J* = 12.5 Hz, 2H); ^{13}C NMR (125 MHz, $CDCl_3$) δ 171.6 (C), 148.7 (CH), 136.0 (CH), 128.6 (CH), 128.4 (CH), 125.7 (CH), 112.6 (C), 102.1 (CH), 90.7 (C), 61.4 (CH_2), 55.2 (CH); IR (KBr, cm^{-1}) 3108,

3080, 2965, 2926, 1743, 1609, 1246, 1136, 1034; HRMS (ESI) *m/z* calcd for $C_{26}H_{20}O_8Na$ $(M + Na)^+$ 483.10504, found 483.10526.

Photoreaction of 1b. The photoreaction conducted according to the procedure outlined above. After 36 h, triphenylmethane (Ph_3CH) was added as an internal standard. ^1H NMR (500 MHz, C_6D_6) was measured to determine the ratio of products. After the photoreaction, the residue was purified by repeated column chromatography and PTLC (hexane : EtOAc = 4 : 1) to give **2b** as colorless crystals. m.p. 137 °C (dec); ^1H NMR (500 MHz, $CDCl_3$): δ 6.66 (dd, *J* = 3.0, 1.2 Hz, 2H), 5.23 (dt, *J* = 3.0, 0.8 Hz, 2H), 5.14 (dd, *J* = 12.5, 0.8 Hz, 2H), 4.27 (dd, *J* = 3.0, 1.2 Hz, 2H), 3.77 (d, *J* = 12.5 Hz, 2H), 1.57 (s, 6H); ^{13}C NMR (125 MHz, $CDCl_3$) δ 173.3 (C), 149.8 (CH), 112.7 (C), 101.3 (CH), 88.1 (C), 61.5 (CH_2), 53.1 (CH), 21.2 (CH_3); IR (KBr, cm^{-1}) 3121, 3113, 2981, 2961, 2931, 1742, 1610, 1241, 1121, 1008; HRMS (ESI) *m/z* calcd for $C_{16}H_{16}O_8Na$ $(M + Na)^+$ 359.07374, found 359.07391.

X-Ray Data of the dimeric product 2a.

Chemical formula	$C_{26}H_{20}O_8$
Formula weight	460.42
Temperature	173(2) K
Wavelength	0.71073 Å
Crystal size	0.05 x 0.20 x 0.22 mm
Crystal system	triclinic
Space group	*P*-1
Unit cell dimensions	a = 9.2883(9) Å alpha = 62.3560(10)°.
	b = 11.2794(11) Å beta = 89.0080(10)°.

	c = 11.6730(12) Å gamma = 89.8930(10)°.
Volume	1083.15(19) $Å^3$
Z	2
Density (calculated)	1.412 Mg/cm^3
Absorption coefficient	0.106 mm^{-1}
F(000)	480
Theta range for data collection	1.97 to 25.00°
Index ranges	-10<=h<=11, -13<=k<=11, -13<=l<=5
Reflections collected	5138
Independent reflections	3721 [R(int) = 0.0125]
Coverage of independent reflections	97.7%
Absorption correction	empirical
Refinement method	Full-matrix least-squares on F^2
Data / restraints / parameters	3721 / 0 / 387
Goodness-of-fit on F^2	0.984
Final R indices [I>2sigma(I)]	R1 = 0.0356, wR2 = 0.1021
R indices (all data)	R1 = 0.0389, wR2 = 0.1060
Largest diff. peak and hole	0.216 and -0.224 e.$Å^{-3}$

^{1}H NMR of **2a**, 500 MHz, $CDCl_3$

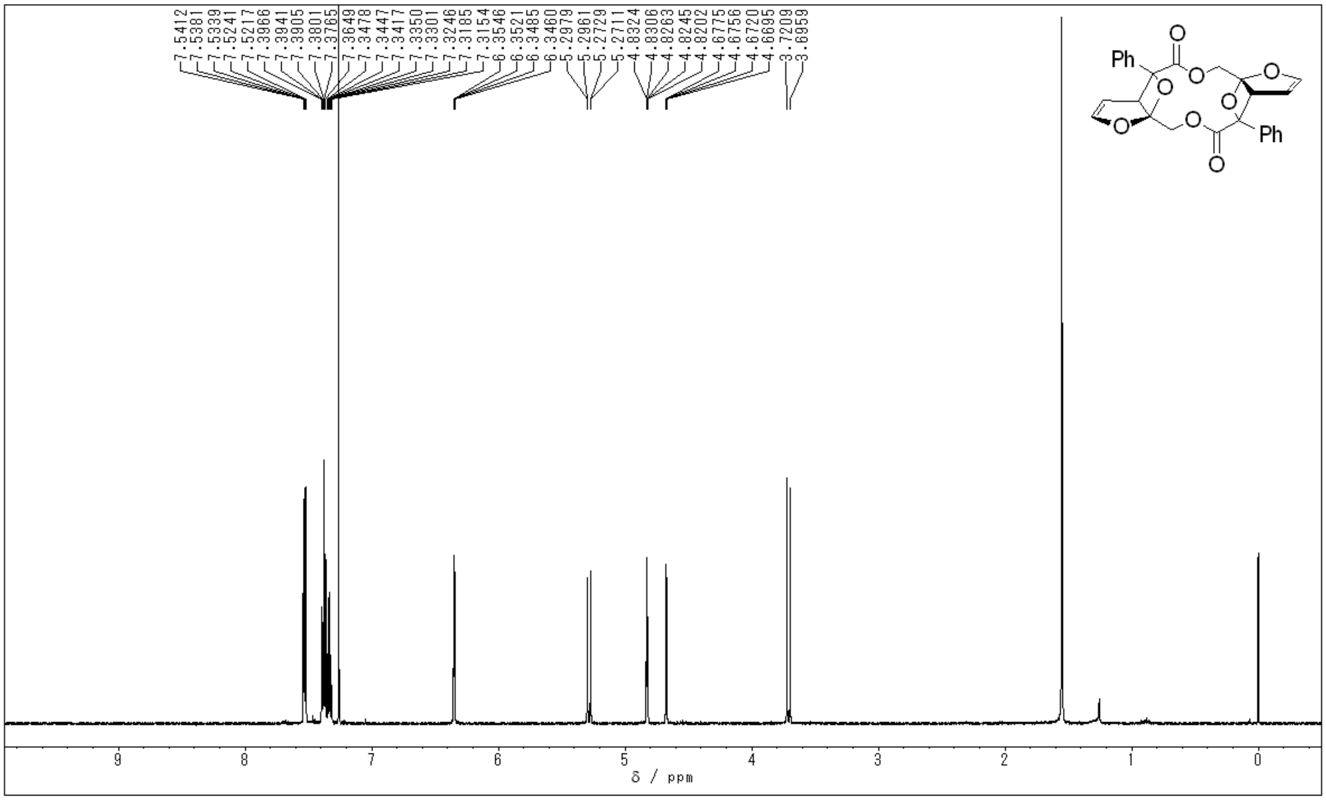

^{13}C NMR of **2a**, 125 MHz, $CDCl_3$ (Upper ^{13}C NMR, Lower DEPT)

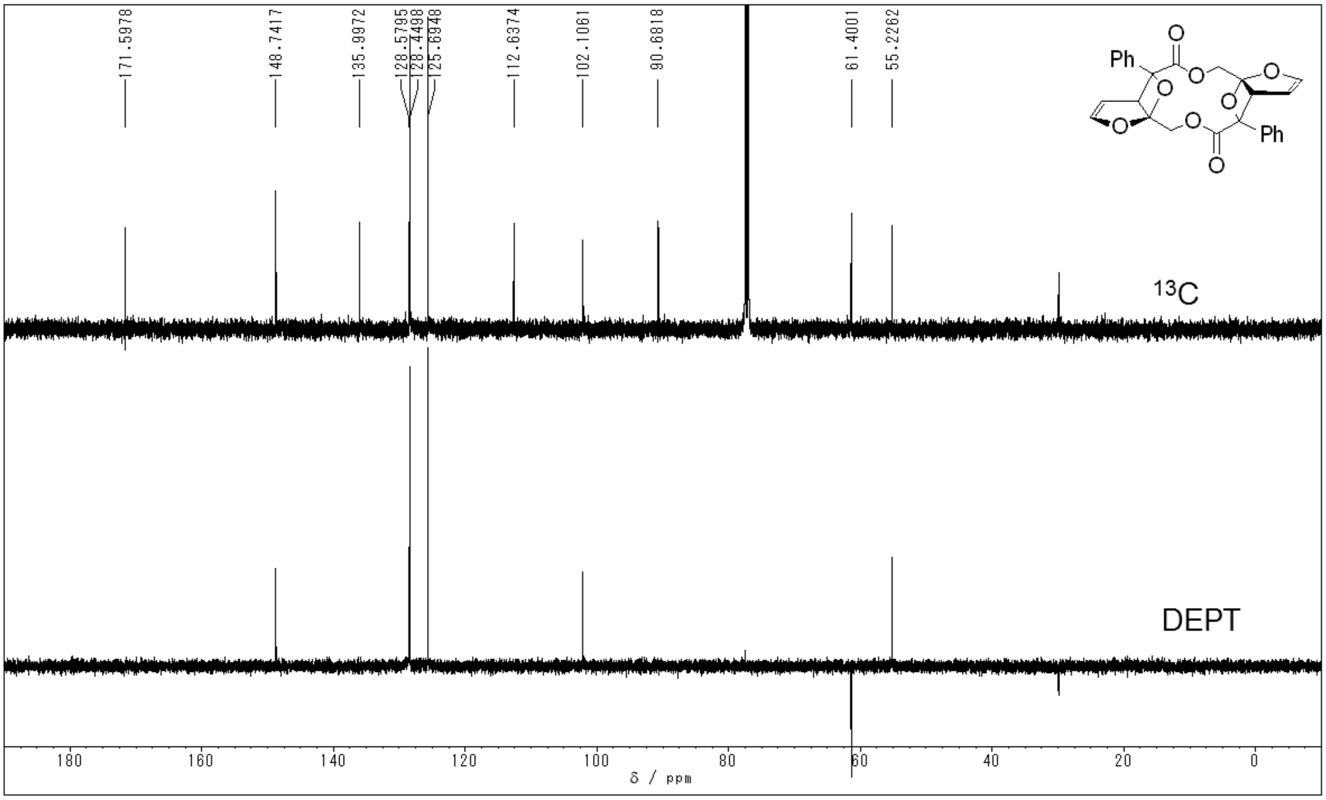

^{1}H NMR of **2b**, 500Hz, $CDCl_3$

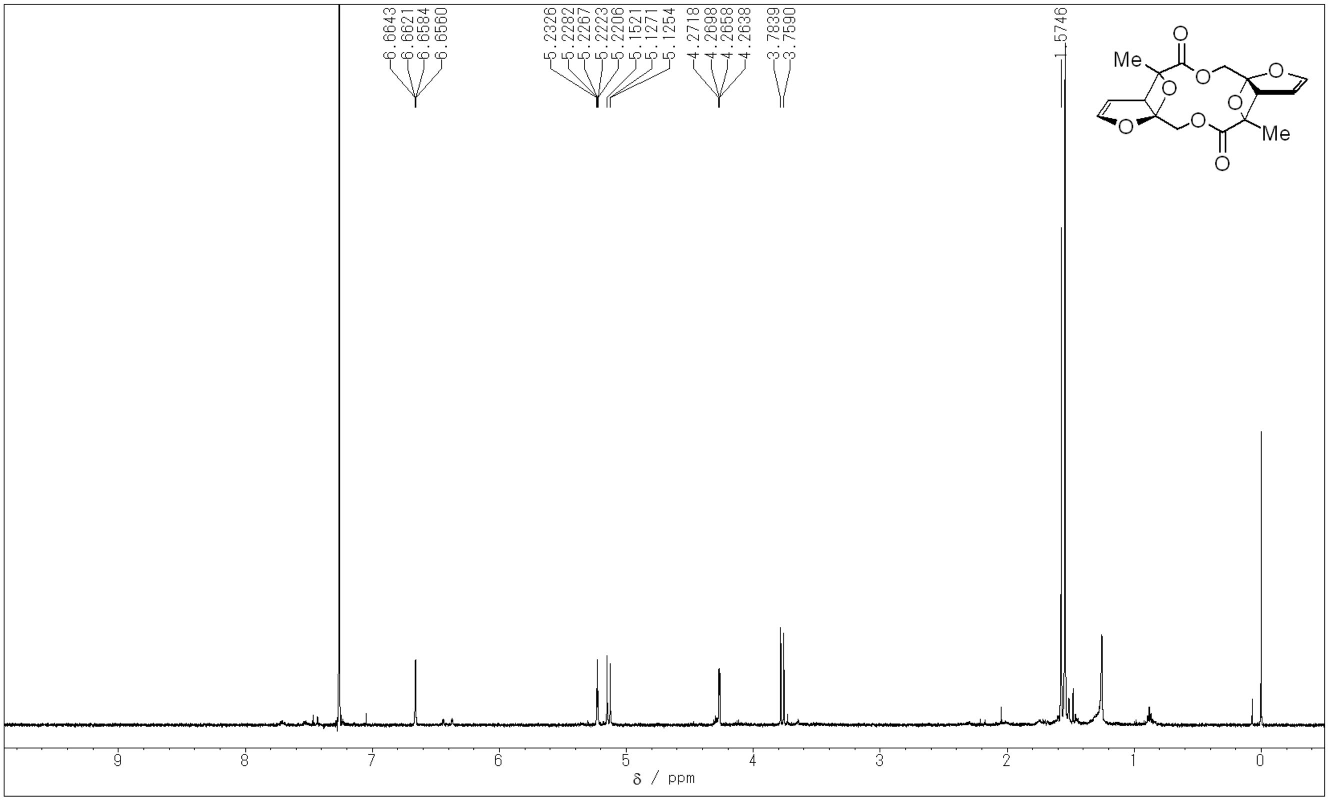

^{13}C NMR of **2b**, 125 MHz, $CDCl_3$ (Upper ^{13}C NMR, Lower DEPT)

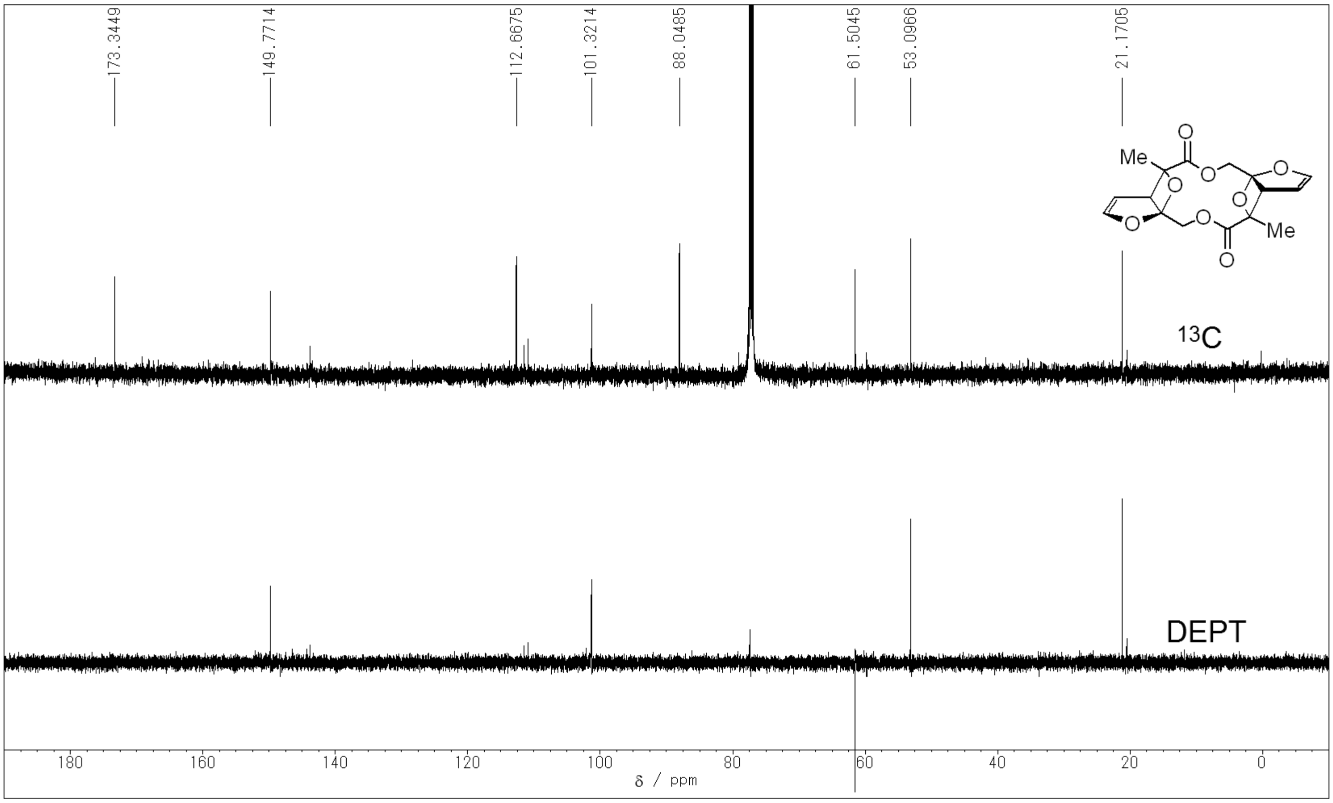

References

[1] Loiseau, F.; Simone, J.-M.; Carcache, D.; Bobal, P.; Neier, R. *Monatsh. Chem.* **2007**, *138*, 121–129.
